# A moisture dynamics perspective on robust changes in characteristics of the tropical circulation under warming

**DOI:** 10.1098/rsos.260963

**Published:** 2026-11-11

**Authors:** Lorenzo Tomassini

**Affiliations:** ^1^ Met Office, Exeter, UK; ^2^ School of Mathematics, University of Leeds, Leeds, UK

**Keywords:** moist convection, atmospheric circulation, moisture dynamics

## Abstract

A process-based view on robust changes in characteristics of the tropical circulation under warming, the intensification of the strongest updrafts and the contraction of the deep-convective ascent area, is presented. The increase in low-level moisture under warming, directly linked to the Clausius–Clapeyron relation, and a positive feedback of latent heating on the dynamics in organized tropical convective systems drives the intensification of the strongest updrafts. Two key sets of negative feedback explain the contraction of the deep-convective ascent area. Increased latent heating in deep-convective ascent areas affects the dry-static stability of the atmosphere and the atmospheric circulation. In addition, the increased latent heating linked to the low-level moisture increase causes enhanced convergence in the deep-convective areas which leads to its dynamic contraction—a process one could call the moisture-dynamical feedback. The changes in the two aspects are related in that both are caused by increased low-level moisture content and subsequent changes in moist processes and moisture dynamics. The formulated moisture-dynamical perspective contrasts with, but does not contradict, an energy balance interpretation of the changes in tropical circulation characteristics in which varying radiative fluxes are attributed an explanatory role.

## 1. Introduction

Two fundamental changes in the characteristics of the tropical circulation under warming emerge in model simulations [1,2] and have in recent years also been emanating to some extentfrom observations [3]. One is the increase of the updraft velocities in the strongest convective storms, the other is the contraction of the area of deep-convective ascent in the tropics. Changes in extreme convective storms have fundamental impacts on livelihoods of communities especially over tropical land [4]. Shifts in deep-convective ascent areas are linked to changes in the migrating tropical rain bands, precipitation margins and monsoons, and have implications for climate and the atmospheric circulation on a global scale.

An important framework for understanding changes in the characteristics of the global climate system under warming is the analysis of the energy budget. Of particular prominence in this context is the so-called radiative feedback analysis, a decomposition of the net heat flux into the climate system into different factors and their individual effect on the top-of-the-atmosphere (TOA) energy budget [5]:

(1.1) N=F−λ⋅ΔT,

where N is the net heat flux into the climate system at the top of the atmosphere, F is the external radiative perturbation in terms of the change in the radiative flux at the top of the atmosphere, λ is the feedback parameter and ΔT the global mean surface temperature change. Assuming linearity, the feedback parameter λ is usually decomposed into individual feedback parameters for different physical processes.

The feedback analysis framework focuses on the radiative effects of various physical processes and uses an energy budget approach to assess their respective influence. An intriguing aspect of equation (1.1) is that formally, as with all budgets, it treats the feedback processes on a par with the forcing term F. This leads to a blurring of the roles of forcing and feedback, of cause and effect. The various physical feedback processes may be seen as *forcing* changes in the TOA energy budget and therefore other climate variations. A prime example is encapsulated in the term *cloud forcing* which describes the TOA radiative effect of the presence of clouds compared to them being absent. Of course, clouds are not an external forcing of the climate system, but the result of a multitude of physical processes which ultimately lead to a radiative effect that influences the TOA energy budget. To consider the radiative effects of clouds as a forcing, or driver, of other processes represents a particular view on the climate system; it is clear that clouds are themselves embedded in a complex environment of interacting processes. Clouds are part of the dynamics of moisture which includes phase transitions and intricate motions on a wide range of scales.

The feedback analysis is an important example of the energy budget approach in climate science and has arguably influenced the way variations in the climate system are conceptualized. In the budget framework, the emphasis is not necessarily on physical processes and mechanisms. Rather, longer-term changes in specific terms are used to explain longer-term variations in other terms of the budget. Which term is explained by which other term is to some extent interchangeable. Moreover, because the TOA energy budget is dominated by radiative fluxes, the feedback analysis highlights the importance of radiative fluxes in that it quantifies all physical processes in terms of their radiative effects. This perspective has been popularized beyond the radiative feedback analysis especially in areas related to moisture and clouds. The role of moisture and clouds is in this context analysed with regard to their radiative effects, and these effects are considered to drive other changes in the climate system, without necessarily considering the convective processes which lead to cloud formation or variations in the humidity distribution. The dynamics of moisture, which includes phase transitions and related temperature and moisture tendencies, is often not seen as a conceptual framework in its own right. Especially in the tropics, clouds are to a large extent directly related to convective processes and the dynamics of moisture. To consider the radiative effects of clouds and moisture as drivers of other changes in tropical dynamics is not incorrect, but it can be questioned whether this is the most appropriate conceptual framework and whether it adequately reflects the order of the most important processes. A more process-based view that emphasizes moist convective processes and puts moisture dynamics at its heart may conclude, in certain circumstances, that the radiative effects of clouds and moisture are more appropriately described as an effect rather than the cause in a chain of processes [6,7].

In [2], the energy budget framework is used to understand the contraction of the ascent area $\alpha_{\text{up}}$ in the tropics under warming; the following derived approximation was used:

(1.2) $$\alpha_{\text{up}}\approx \frac{1}{1+\frac{Q_{\text{up}}}{Q_{\text{down}}}},$$

where $Q_{\text{up}}$ is the diabatic (latent and radiatve) heating in the ascent area, and $Q_{\text{down}}$ is the diabatic heating in the area of descending air. Equation (1.2) assumes that mass is conserved over the considered area and that there is no exchange of mass between the tropical and extra-tropical regions. The core of the argument is that $\alpha_{\text{up}}$ decreases under warming because $Q_{\text{down}}$ increases more slowly than $Q_{\text{up}}$. In areas of ascent, latent heating dominates over radiative heating, and $Q_{\text{down}}$ represents mainly radiative longwave (LW) cooling in areas of subsidence. In somewhat simplified terms, neglecting the effect of changes in radiative heating in ascent areas, one could therefore summarize the argument by saying that the tropical ascent area contracts because the latent heating in ascent areas increases faster than the LW radiative cooling in areas of descent.

The argument is valid and interesting. One might ask, however, to what extent this can be considered an explanation. There is not really a fundamental theory of how LW cooling changes under global warming, for example. Sometimes it is implicitly suggested, as in [8], that the change in LW cooling under warming is due to the fundamental radiative properties of water vapour and cloud liquid water. But the change in LW cooling, even assuming clear-skies, is the result of a multitude of thermodynamical and dynamical process and does not follow directly from basic radiative properties of the water molecule [9,10]. The right-hand side of equation (1.2) is essentially as empirical as the left-hand side, a result of the fact that equation (1.2) is ultimately derived from an energy budget.

In this article, the usefulness of an energy budget view on tropical circulation changes is not questioned, but it is argued that an alternative perspective that centres around moisture dynamics and moist diabatic processes provides a more process-based conceptual framework for understanding these shifts in circulation characteristics. It can help to focus the attention on the most relevant physical mechanisms and might more adequately distinguish between causal relations and longer-term adjustment processes. Processes related to clouds and convection happen on relatively short time scales of hours to days, and on those shorter time scales moist diabatic processes are often more important in shaping the local and regional circulation characteristics than radiative processes. In that sense, from a perspective of a mechanistic understanding, it can be more appropriate to consider radiative effects and related terms in the energy budget as adjusting to fundamental moist processes rather than the other way around. The Clausius–Clapeyron relation, which describes the variation of saturation specific humidity with temperature and pressure, is an example of a basic physical principle directly linked to moisture which plays an important role in shaping convective processes, clouds and related circulations.

We first briefly stand back and reflect on the notions of cause and effect in complex systems such as the Earth’s atmosphere and climate system (§2). In §3, the contraction of deep-convective ascent areas and the increase in strong updrafts in the tropics under warming are reviewed looking at several different datasets such as ERA5, global kilometre-scale resolution warming experiments and idealized radiative–convective equilibrium (RCE) warming experiments. Idealized RCE sensitivity experiments are then used to investigate the role of changes in latent heating and related moisture dynamics in the variation of tropical circulation characteristics (§4). We finish with a discussion and conclusions in §5.

## 2. A reflection on cause and effect in complex systems

When reflecting on explanations or causes for changes in characteristics of the climate system, a basic, almost philosophical, issue is the question: what can be considered a cause and what is more appropriately addressed as an effect or an interaction? The Earth’s climate is a complex system with manifold processes influencing each other on a wide range of spatial and temporal scales. Strictly speaking, only factors such as the incident solar radiation and maybe the atmospheric composition can be considered external drivers of the climate system. All other processes are internal and interact with each other in a multitude of ways. Therefore, in a strict sense, not one process is purely the cause of the other. Nevertheless, in the context of better understanding a particular problem, it can be useful to try to identify the main physical processes that primarily and most directly affect a certain phenomenon.

The consideration of budgets, such as the water or energy budget, is useful in the study of complex systems because they formulate quantitative constraints that relate different terms of the budget with each other even though they do not necessarily imply causality in a narrow sense. A complete set of budget equations may define the governing equations of the system. Thus, there is not really a clear distinction between a budget equation and a causal relationship. Nevertheless, for a given process in the climate system, specific other processes might arguably be called the immediate and primary causes, more so than a set of alternative mechanisms.

In order not to get lost in semantics, it is useful to look at a concrete example such as the formation of convective clouds. It suggests itself to consider the processes that lead to buoyancy, then condensation and finally the development of a cloud as causes for the formation of the cloud. On the time scale of the cloud formation, radiative processes usually play less of a role and have a weaker influence. There is an important counterexample, namely the formation of stratocumulus clouds which are to a large part driven by cloud-top radiative cooling. But for other convective cloud types, such as the evolution of deep cumulonimbus clouds, radiative effects are typically less important even though the radiative effect of the cloud plays an important role in the TOA energy budget and longer time-scale processes.

Time scales play a role when thinking about causality in complex systems. For example, slowly evolving sea-surface temperatures influence where clouds form, but their variability is often not the immediate cause of the formation of a particular cloud which happens on shorter time scales. In climate science, typically long-term mean quantities are analysed, a fact that hampers the appreciation of the underlying physical processes. In particular, in budget equations, the considered terms are usually means over relatively long time scales that do not necessarily correspond to the time scales of the investigated process in question. Terms in a budget, such as the energy budget, can be the result of longer-term adjustments, not immediate causes. The analysis of quantities averaged over long, climatic time scales makes it harder to identify cause and effect. So-called process studies often choose to consider shorter time scales. Of course, this depends on the process in question. Some processes exhibit long time scales, such as the uptake of heat in the deep ocean. In that case, it is appropriate to consider the integral of a wide range of time scales.

Another issue with budgets can be that they are sometimes designed specifically to be invariant under a certain process and, by providing a theoretical framework for analysing a complex system with many interacting mechanisms, tend to emphasize the terms that explicitly occur in the budget equation. Then, a superficial interpretation of the budget might conclude that the invariant quantities are less important. An example here is moist static energy (MSE) which is constructed in such a way that condensation and the release of latent heat do not change MSE. The MSE perspective thus emphasizes the processes that provide the MSE to the atmosphere such as TOA radiative fluxes or surface fluxes. Nevertheless, with regard to the creation of available potential energy (APE) and related circulations, for instance, the condensation process is an important aspect and the fact that it does not change MSE does not imply its insignificance. It is these circulations induced by latent heating that in turn affect the surface fluxes and, through moisture dynamics and cloud formation, the radiative fluxes.

We do not intend to dive deeper into a philosophical discussion on the concepts of cause and effect. Relatively simple criteria such as how directly and pronouncedly a process affects the other, how plausible a physical mechanism is or how the respective time scales are related can give guidance on what can usefully be called a cause in a certain context when investigating complex systems with many interacting processes.

## 3. The contraction of deep-convective ascent areas and the increase in strong organized storms in the tropics under warming

We aim at studying the contraction of the deep-convective ascent area under warming in the tropics and the intensification of the strongest updrafts in organized convective storms from a process-based point of view. The dynamics of moisture, which includes the release of latent heat by condensation, is closely related to these changes in circulation characteristics.

The intensification of the strongest upward vertical velocities can also be formulated as the increase in the area of the strongest updrafts at any point in time. This way the change in frequency of occurrence of strong-convective events is equivalent to the change in the area where these strong storms occur. In order to deal with a clearly defined problem, the areas of strong convective storms and the deep-convective areas are defined here as the extent of the areas where the vertical velocities exceed particular, absolute thresholds. The exact threshold values can vary depending on the temporal and spatial resolution of the considered data, but the strongest convective storms are defined as exceeding a very high threshold in terms of their vertical velocities, and the deep-convective areas are defined as exceeding a modest threshold value. The expansion of the strong-convective areas and the contraction of the deep-convective areas are thus defined based on the changes in the tropical circulation (more precisely, the changes in the distribution of vertical velocities) under warming, not changes in rainfall. Even though the changes in the circulation characteristics are quite closely linked to changes in rainfall, the two aspects are not the same and can differ because changes in rainfall are also directly influenced by alterations in the moisture content of the atmosphere.

A range of different datasets is considered in the following, from reanalysis data to global kilometre-scale resolution simulations and idealized modelling experiments. Circulation features are difficult to observe directly and we start by investigating the evolution of vertical pressure velocity in ERA5 reanalysis over the historical period.

### 3.1. Vertical pressure velocities in ERA5

Tropospheric vertical velocity is not available in the ERA5 reanalysis [11]; the vertical pressure velocity at 500 hPa is considered instead. Even though vertical pressure velocity is not the same as vertical velocity and bears a relation to mass flux, it still gives a reasonable indication of interannual changes in the mid-tropospheric circulation. The tropics are defined to be located between 30${}^{\circ }$ South and 30${}^{\circ }$ North and variations in annual means are computed for the period 1974 to 2024 (figure 1). To calculate deep-convective ascent areas, a modest threshold of 0.5 Pa s^–1^ is assumed; for strong-convective ascent areas, a high threshold of 3.5 Pa s^–1^ is taken as a basis. The ascent areas are determined for every six-hourly time step and then the annual means of those areas are computed. The areas are given as percentage of the total area of the tropics. In figure 1, these tropical ascent area fractions are normalized by their respective time means and standard deviations in order to be able to present all the lines in the same figure.

**Figure 1.**
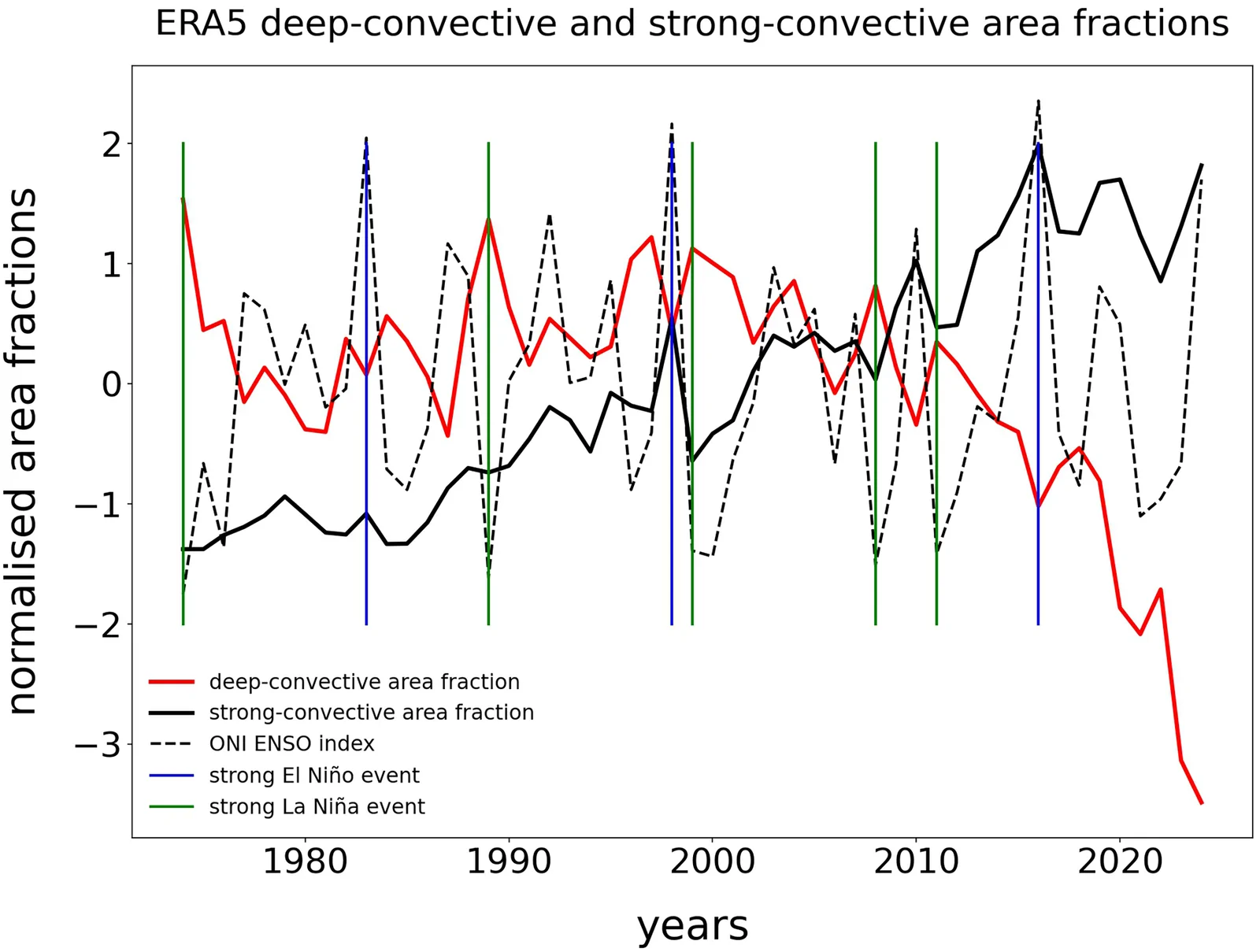
Standardized deep-convective area fraction (red line) and standardized strong convective area fraction (black line) based on ERA5 vertical pressure velocity at 500 hPa. The dashed line represents the Oceanic Niño ENSO index. Vertical lines indicate strong El Niño events (blue lines) and strong La Ninã events (green lines). The time series of tropical ascent area fractions have been normalized by their respective time means and standard deviations in order to be able to present all the lines in the same panel.

Over the historical period of 1974 to 2024, which broadly agrees with the satellite era, the reanalysis data confirm that the deep-convective ascent areas (red line) have contracted and the strong-convective areas (black line) have expanded. In the tropics, the interannual variability is most strongly influenced by the El Niño Southern Oscillation (ENSO) mode of variability, and the relationship of the ascent areas to the Oceanic Niño Index (ONI) is interesting to observe (dashed line). There is quite convincing evidence that ENSO is anti-correlated with the deep-convective ascent area fraction and positively correlated with strong-convective tropical area fraction, at least for the strongest ENSO events indicated by blue (positive ENSO) and green (negative ENSO) vertical lines in figure 1. In the tropical mean, positive ENSO events tend to be warmer and negative ENSO events cooler in terms of the near-surface temperature.

The evolution of the ascent areas is not linear over time and there are uncertainties around the accuracy of the ERA5 pressure velocity, but at least the reanalysis data confirm the qualitative behaviour of the deep-convective and strong-convective ascent areas, namely a contraction and an expansion, respectively. As explored in the next section, the pattern of warming also influences the values of the ascent area fractions, and a more detailed understanding of their historical behaviour would need to consider various different modes of climate variability.

### 3.2. Tropical circulation changes in global kilometre-scale resolution warming experiments

The global kilometre-scale resolution warming experiments presented and discussed in [12] confirm the general observation that under warming the deep-convective ascent area contracts and the strong-convective area expands (table 1). The experiments involve a global 5 km resolution model with prescribed sea-surface temperatures that is used to simulate the historical year 2020. Two different warming patterns are added to the historical, daily varying sea-surface temperatures of the year 2020, a La Niña-like pattern and an El Niño-like pattern. For the deep-convective ascent area, a modest threshold of 0.5 m s^–1^ is used, and for the strong-convective ascent area, a high threshold of 3.5 m s^–1^ is employed.

**Table 1.** Deep-convective and strong-convective tropical ascent area fractions for the historical year 2020 and the two global kilometre-scale patterned warming experiments. The tropical area fractions are computed at each six-hourly time step, and then the mean is calculated over all time steps.

experiment	deep-convective area fraction	strong-convective area fraction
historical year 2020	0.017955	8.99492e-05
La Niña-like warming	0.016543	0.00022221
El Niño-like warming	0.017709	0.00014404

The La Niña-like warming pattern shows a stronger contraction of the deep-convective ascent area than the more uniform El Niño-like warming pattern. And analogously, the expansion of the strong-convective ascent area is more distinct in the case of the La Niña-like warming pattern experiment than the El Niño-like counterpart. As discussed in [12], the more pronounced strengthening of the Intertropical Convergence Zones (ITCZ) and the intensification of the moisture transport into these regions in the La Niña-like warming simulation are probably a main part of the explanation.

### 3.3. Radiative–convective equilibrium warming experiments

The RCE model experiment is an idealization of the near-equatorial tropical atmosphere. The experimental layout of RCE simulations is typically based on prescribed uniform sea-surface temperatures, no land, constant incoming solar radiation and the absence of rotation—assumptions that are valid to first order in the tropics near the Earth’s equator in particular. The simple experimental set-up of RCE allows for conducting a variety of sensitivity experiments relatively easily and for exploring the behaviour of tropical convection. A basic sensitivity experiment concerns the response of tropical convection to a warming of the sea surface.

All the simulations presented here were run for 3 years, and the last month of the data was used for the analysis. The experiments were part of and follow the protocol of the intercomparison study by Wing [13]. The experimental set-up is identical to what is called *RCE_large* with the global model *UKMO-GA7.1* in [13]. The vertical velocity at 500 hPa has hourly time resolution. Since the RCE experiments discussed here were conducted at relatively low resolution, the thresholds used to define deep-convective and strong-convective area fractions are different from the ones considered in previous sections, namely 0.03 m s^–1^ and 0.1 m s^–1^ for hourly mean vertical velocity at 500 hPa, respectively. The area fractions are computed for every hourly time step and then the mean is determined over all time steps.

RCE sensitivity experiments are analysed in more detail in the next section. For now, we can note that under warming sea-surface temperatures, deep-convective area fractions contract and strong-convective area fractions expand (table 2), consistent with the results from previous sections based on reanalysis data and the global kilometre-scale warming experiments. The responses are monotonic in the prescribed surface temperatures, but not linear.

**Table 2.** Area fraction of vertical velocity at 500 hPa exceeding defined thresholds for RCE experiments with 295 K, 300 K and 305 K prescribed sea-surface temperatures. This is computed at each time step, and then the mean is calculated over all time steps of one month at the end of the 3-year simulation.

experiment	deep-convective ascent area fraction	strong-convective ascent area fraction
295 K	0.0564	0.00061
300 K	0.0455	0.00116
305 K	0.0430	0.00432

## 4. Radiative–convective equilibrium latent heating sensitivity experiments

To explore physical processes underlying the changes in tropical circulation characteristics under warming, a series of RCE sensitivity experiments is investigated in which the specific latent heating from condensation is varied. The rationale for these experiments is to better understand the role of latent heating and more generally the dynamics of moisture in changes of tropical deep-convective and strong-convective area fractions. The advantage of the sensitivity experiments is that they allow for prescribing controlled changes which makes the observed differences between experiments attributable to a particular cause. As touched upon in §1, in energy budget approaches, the importance of radiative and surface fluxes is often emphasized in this context. Here, a more process-based view on these changes in circulation characteristics is explored, centred around the significance of latent heating in determining features of the atmospheric circulation in the tropics.

### 4.1. The latent heating sensitivity experiments

The standard experiment is the RCE simulation with the sea-surface temperature prescribed at 300 K and using a value of $2.501\times \;10^{6}$ J kg^–1^ for the specific latent heat of condensation for water L in the model. This reference experiment is compared to five sensitivity experiments in which the specific latent heat of condensation is varied. Figure 2 shows snapshots of outgoing LW radiation (OLR) for the different experiments at the end of the 3-year-long integrations.

**Figure 2.**
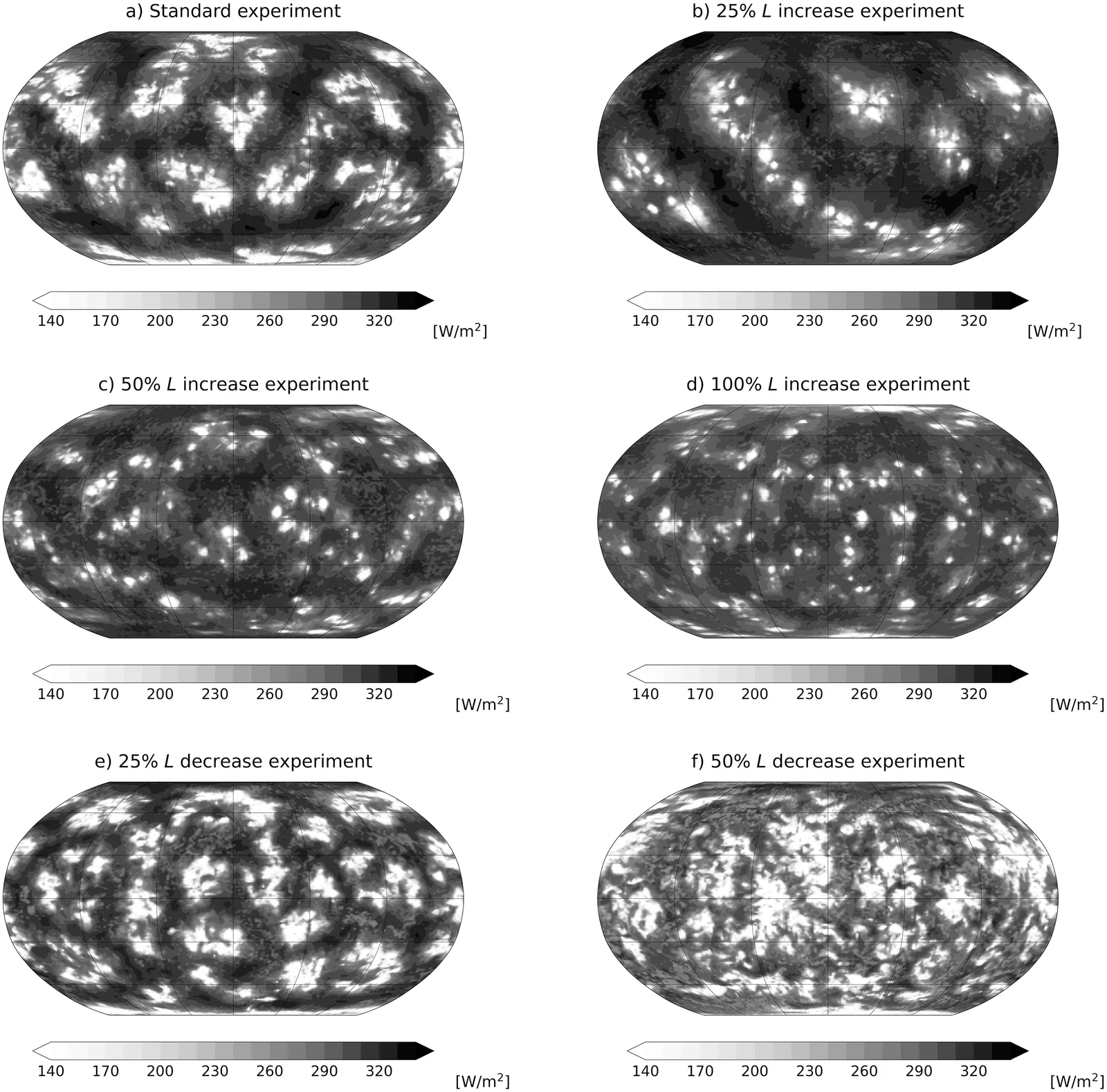
Outgoing LW radiation (OLR) in the experiments with changed specific latent heating of condensation. Panel (a): standard experiment, panel (b): 25% increase in specific latent heat of condensation, panel (c): 50% increase, panel (d): 100% increase, panel (e): 25% decrease and panel (f): 50% decrease.

What is immediately obvious is that the specific latent heat of condensation L has a strong impact on the deep-convective cloud structure. In particular, the deep-convective cloud area characterized by low values of OLR strongly contracts with increasing L. The changes in the deep-convective and strong-convective area fractions in the different experiments are, however, not entirely straightforward (table 3). Generally, the deep-convective areas decrease with increasing L and the strong-convective area fractions increase, but the changes are not always monotonic, indicating that various feedbacks play a certain role. For example, the deep-convective areas contract more with a 25% increase in L than with a 50 or 100% increase. Note too, that the changes in strong-convective areas are not strictly monotonic in L.

**Table 3.** Area fractions of vertical velocity at 500 hPa exceeding defined thresholds for RCE experiments with 300 K prescribed sea-surface temperature and varied specific latent heat of condensation. This is computed at each time step, and then the mean is calculated over all time steps of one month at the end of the 3-year simulation.

experiment	deep-convective ascent area fraction	strong-convective ascent area fraction
standard	0.0455	0.00116
25% increase	0.0212	0.01496
50% increase	0.0275	0.01629
100% increase	0.0259	0.01424
25% decrease	0.0602	0.00134
50% decrease	0.0972	0.00177

The structures of the vertical velocities and the specific humidity are consistent with what can be observed in terms of OLR. The areas of strong updrafts are indeed much narrower in the doubled specific latent heat experiment with moist areas around them (figure 3). In the free troposphere, the double L experiment is generally moister than the standard simulation (figure 4), mostly because the middle troposphere is warmer due to the stronger latent heating (see figure 5). Thus, the moist areas are not narrower and the low-level specific humidity gradients are not enhanced in the double L experiment. The same is true for relative humidity (not shown).

**Figure 3.**
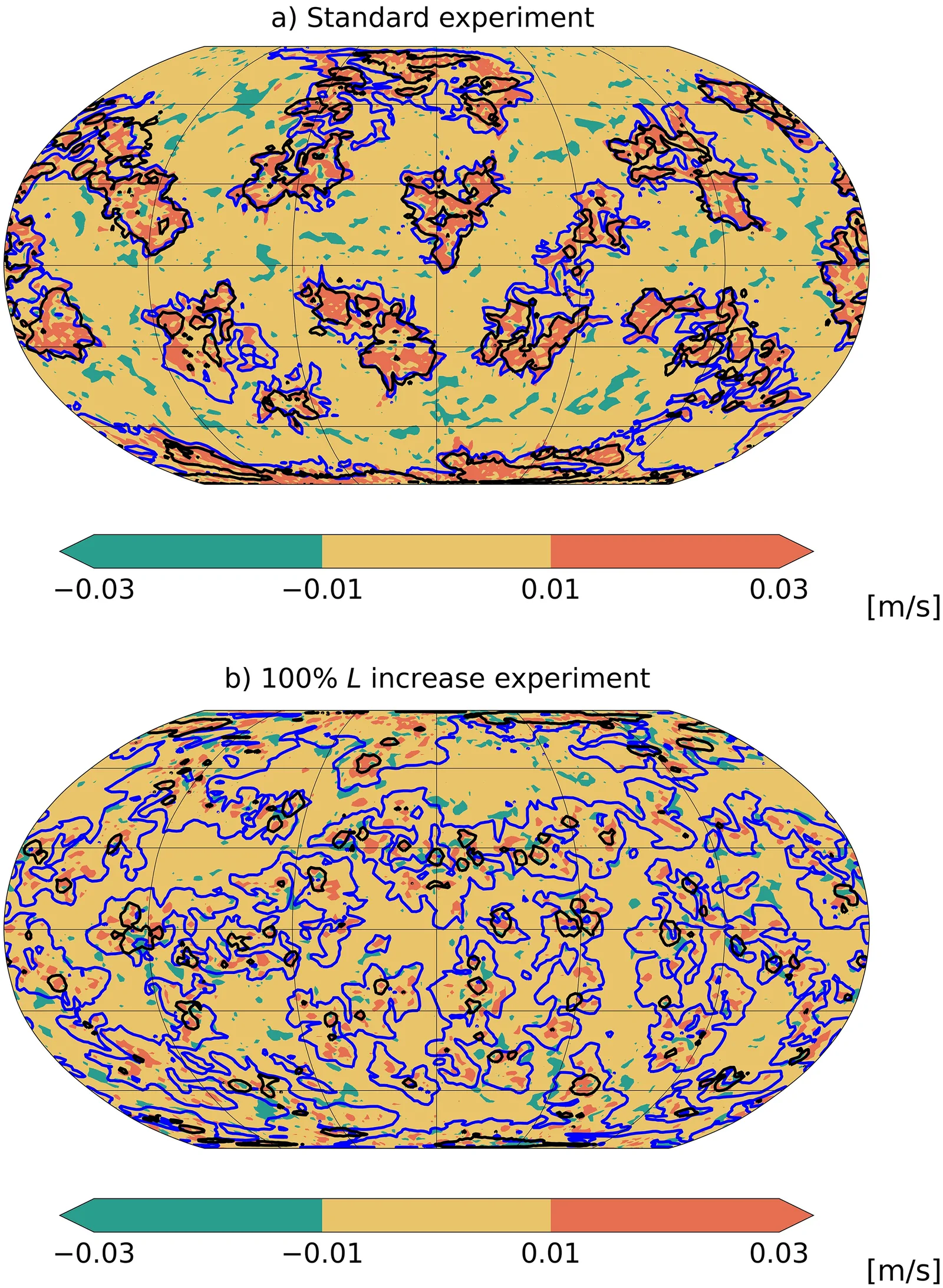
Vertical velocity (colour shading) and specific humidity (blue contour) at 1400 m height for the standard experiment (top panel) and the 100% latent heat increase experiment (bottom panel). Regions of strong upward vertical velocities coincide with moist patches. Areas of deep-convective vertical velocities above a threshold of 0.01 m s^–1^ contract in the increased latent heating experiment. The specific humidity contour is 11 g kg^–1^. The black lines indicate the 220 W m^–^${}^{2}$ contour of OLR to facilitate comparison with figure 2a,d, respectively.

**Figure 4.**
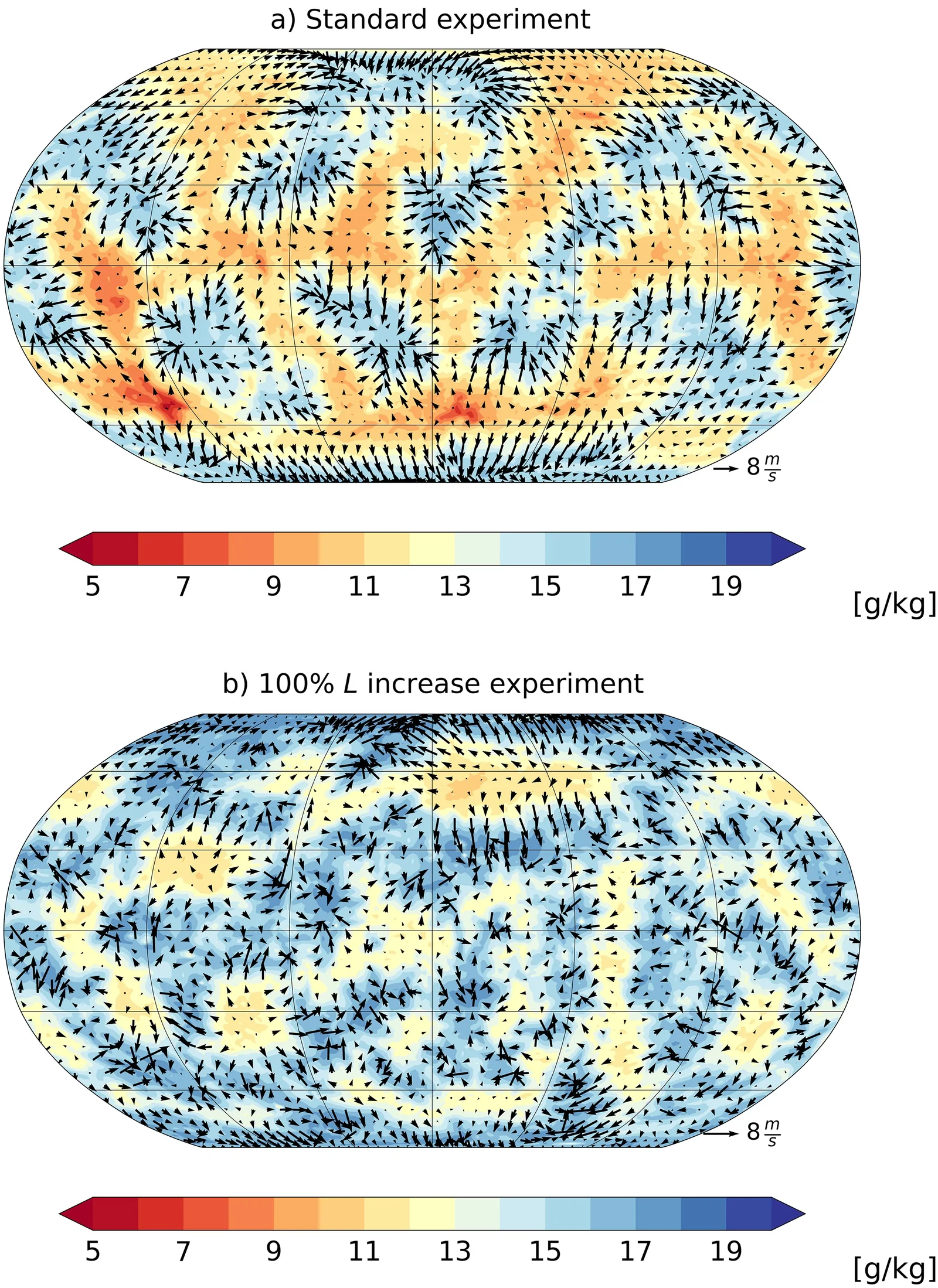
Specific humidity (colour shading) and winds at 275 m for the standard experiment (top panel) and the 100% latent heat increase experiment (bottom panel). The 100% latent heat increase experiment is generally moister than the standard experiment mainly because it is warmer in the middle troposphere (figure 5). Because all regions are moister, it is not primarily the moisture gradients that increase in the 100% latent heat increase experiment; the low-level moisture convergence in deep-convective ascent areas, however, is markedly enhanced in the double L simulation.

**Figure 5.**
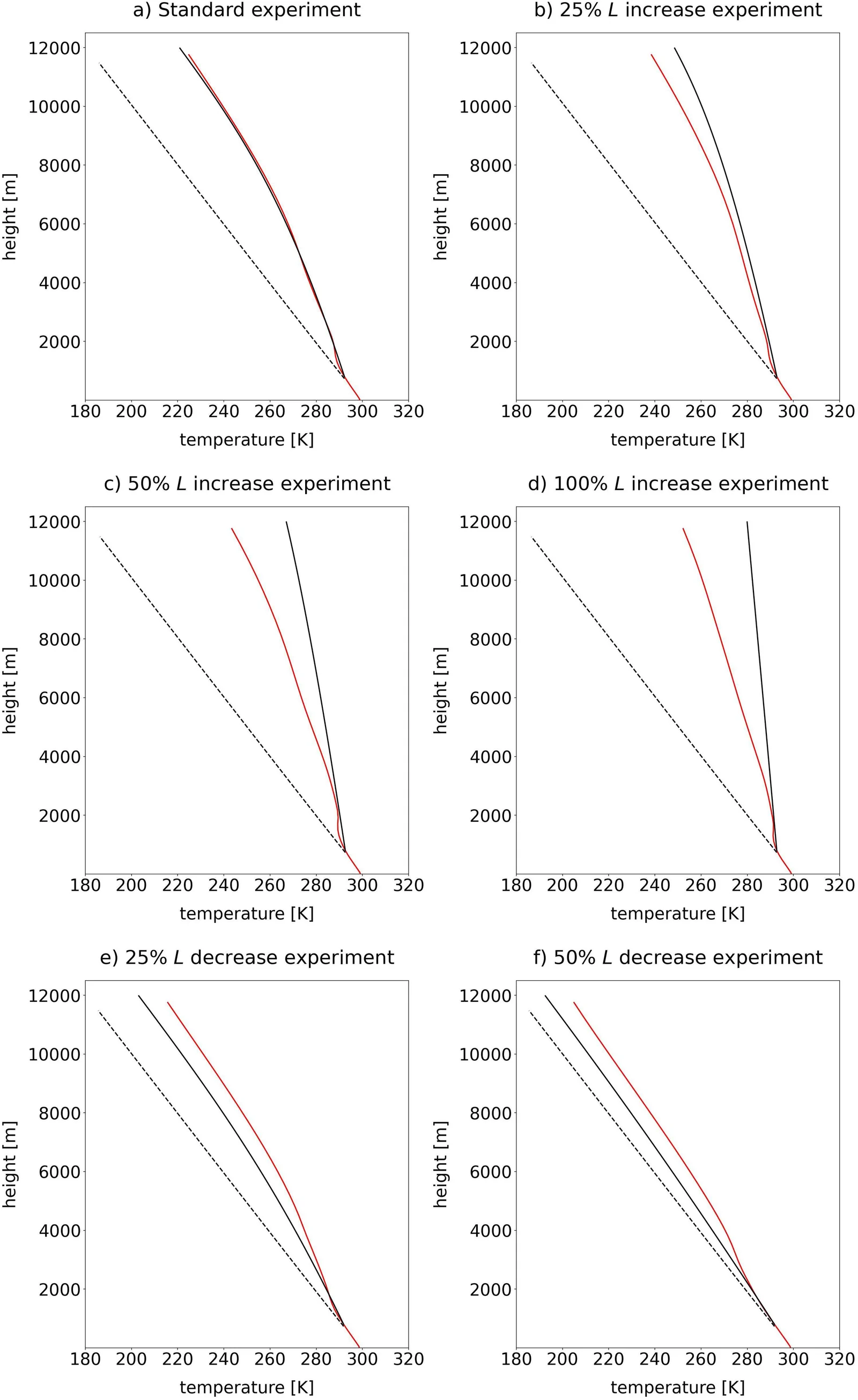
Vertical profiles of global mean temperature in the experiments with changed specific latent heating of condensation (red lines). Panel (a): standard experiment, panel (b): 25% increase, panel (c): 50% increase, panel (d): 100% increase, panel (e): 25% decrease and panel (f): 50% decrease. The moist adiabats (black lines) are calculated using the corresponding values for the specific latent heating of condensation. The dashed lines indicate the dry adiabat.

With increasing L, the moisture convergence into the deep-convective areas becomes stronger, the middle troposphere gets warmer and moister around the deep-convective updrafts. Even though generally the deep-convective areas contract with increasing L and strong-convective areas expand, the changes are not linear in L and there are indications that secondary feedbacks play a role (table 3). In addition to the increased low-level convergence in convective ascent areas caused by the stronger latent heating, the resulting changes in atmospheric stability are an important aspect which affect the characteristics of the clouds and vertical velocities. One should stress, however, that by design of the experiments the ultimate physical *cause* of all the changes seen in the different sensitivity experiments is the changed specific latent heat of condensation.

### 4.2. The role of low-level moisture convergence

The response of the atmospheric circulation to the latent heating and the resulting low-level moisture convergence is central to the contraction of the deep-convective areas. The low-level moisture convergence sustains the latent heating in the deep-convective areas. This is a positive feedback that one can call a moisture-dynamical (or moisture-circulation) feedback since both the presence of moisture and the atmospheric circulation are essential. This moisture-dynamical feedback is absent in regions where no latent heating occurs, and it is the main reason why there is an asymmetry between the extent of the areas of convective motion and the extent of areas of subsidence even in simple models of moist convection with uniform surface temperatures [14,15]. Convective areas are typically more confined than the regions of downward motion. The stronger the latent heating the more pronounced the asymmetry is, and therefore an increase in the specific latent heat of condensation leads to a contraction of the deep-convective areas.

A mechanistic description of the process would set out how increased latent heating leads to an atmospheric circulation response with stronger upper-level divergence and lower-level convergence. The enhanced low-level convergence feeds moisture into the deep-convective areas, which promotes more latent heating. This chain of events constitutes a positive feedback which is absent in regions of downward motion and it is the main reason why there is an asymmetry between the extent of the areas of convective motion and the extent of areas of subsidence, including in the real tropical atmosphere.

In the context of the same argument, one may also invoke a mass budget perspective. If the latent heating is increased, then the upward vertical velocities are enhanced as a consequence. Because the mean vertical velocity (strictly speaking, the mean mass flux) is zero or close to zero over the whole domain owing to mass continuity, the area of deep-convective upward motion tends to contract. But as with all budget arguments, this is not a strictly mechanistic argument because the total upward mass flux could increase in principle; all the terms in a budget can change until a new balance is reached. A budget formulates a balance and a relation between different terms, not necessarily a physical mechanism. In 1938, Jacob Bjerknes discussed the interplay between latent heating, the atmospheric circulation and the ratio between updraft and subsidence areas in cumulus convection based on a simple model [14]. Increased condensational latent heating causes enhanced rising motion in cloudy regions. Given a mean vertical velocity, the increased vertical velocity in the convective areas can be achieved by making the areas of rising motion narrower. Later, other researchers extended this argument along similar lines [15–17].

The variation in low-level MSE gradients across the different sensitivity experiments is an important feature that is related to the strength of low-level convergence into the deep-convective areas (figure 6). The intensity of the low-level convergence into the deep-convective areas increases with increasing L (figure 7). This is also confirmed by the values in table 4 which lists mean values of the deep-convective and strong-convective updrafts for the different experiments. The vertical velocities in both deep-convective and strong-convective ascent areas get stronger in the increased L simulations. Both the modest and the strongest updrafts intensify, but the areas of the modest updrafts contract, while for the very strongest updrafts, this implies an expansion of their area (figure 7). The mechanism is similar to the process underlying the contraction of the ITCZ described by Byrne & Schneider [1] and Lau & Kim [18]. In [1], it is discussed how the main tendency for the contraction of the ITCZ comes from the strengthening of the meridional MSE gradient under warming; an MSE budget approach is adopted, which somewhat underemphasizes the role of latent heating in deep-convective updrafts and the circulation changes it causes because latent heating is not a term occurring explicitly in the MSE budget.

**Figure 6.**
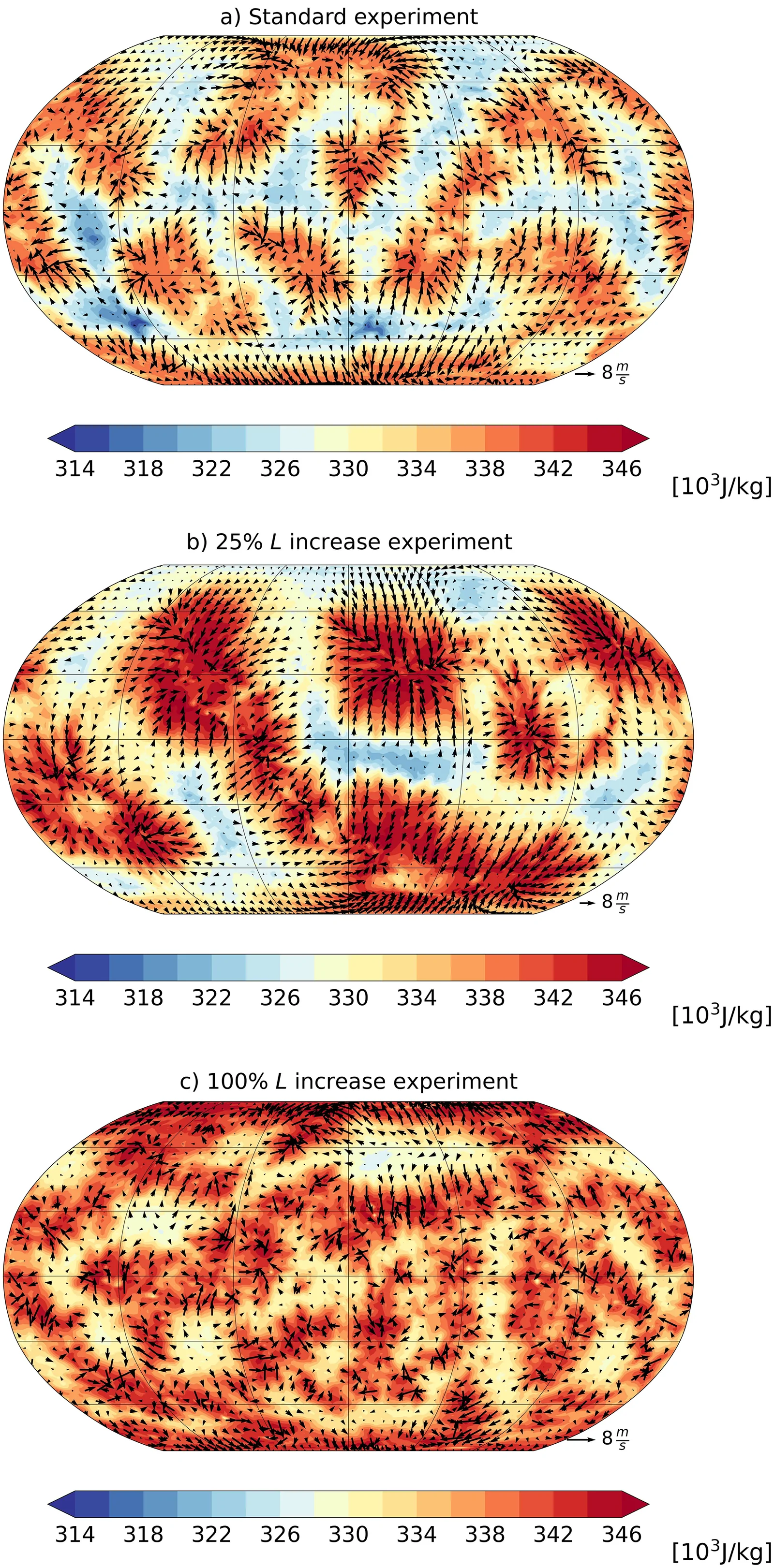
MSE and winds at 275 m height for the control experiment, the 25% increase experiment and the 100% latent heat increase experiment. The pronounced contraction of the ascent areas in the 25% increase experiment is partly due to the distinct increase in horizontal MSE gradients and resulting convergence in deep-convective ascent areas.

**Figure 7.**
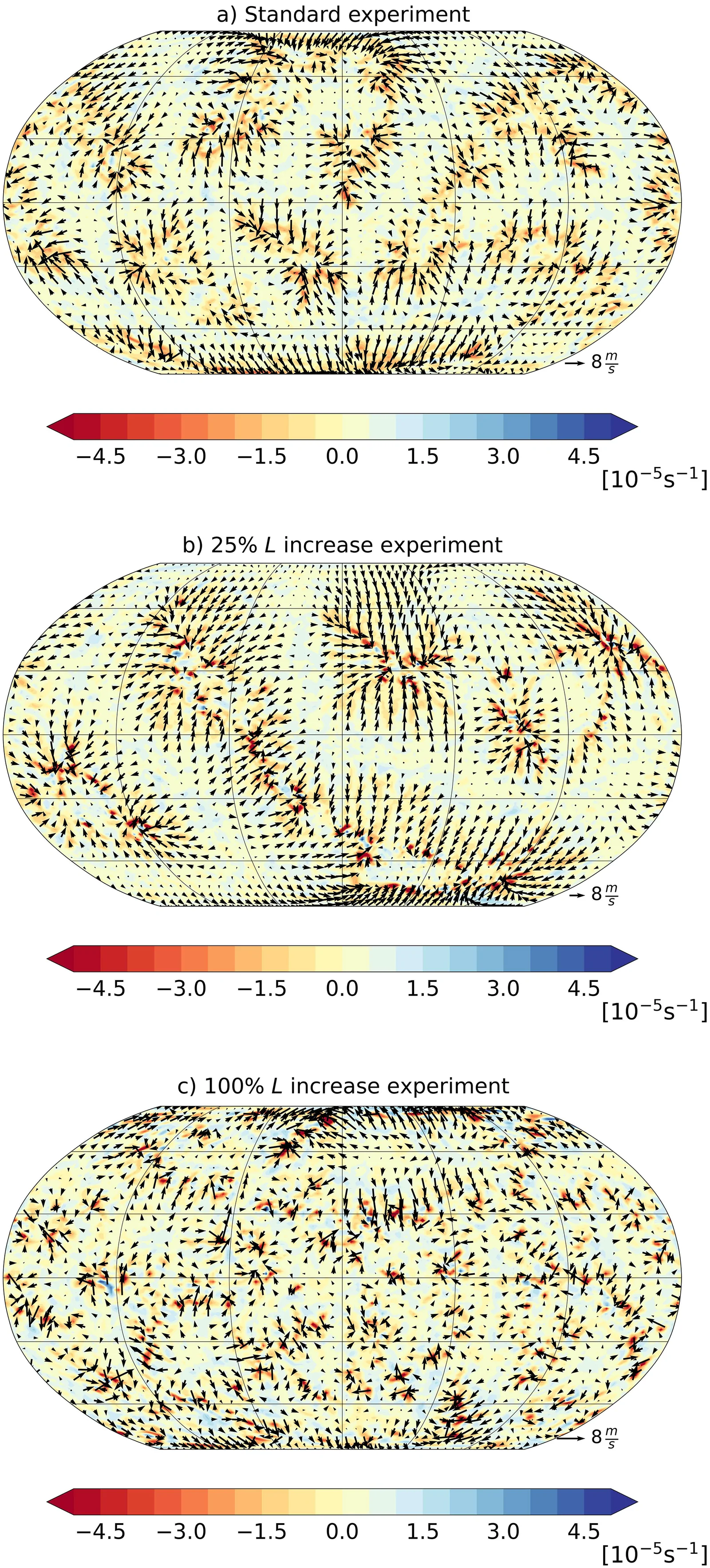
Divergence and winds at 275 m height for the control experiment, the 25% increase experiment and the 100% latent heat increase experiment. Convergence in deep-convective ascent areas in the 100% increase experiment is enhanced more modestly than in the 25% increase experiment.

**Table 4.** Mean values of vertical velocities at 500 hPa for those velocities that exceed the defined thresholds. This is computed at each time step, and then the mean is calculated over all time steps of one month.

experiment	deep-convective velocities [m s^–1^]	strong-convective velocities [m s^–1^]
standard	0.05006	0.11689
25% increase	0.17179	0.21884
50% increase	0.13804	0.19154
100% increase	0.12205	0.17077
25% decrease	0.05086	0.11412
50% decrease	0.05119	0.11169

It is not global warming that is investigated in the sensitivity experiments here, but latent heating when and where deep convection occurs is expected to increase under global warming, too, because low-level moisture increases approximately according to the Clausius–Clapeyron relation. The situation discussed in the RCE sensitivity experiments is easier because it is known what the cause of the observed changes is, namely the varied specific latent heat of condensation. In the RCE, experiments increased latent heating, causing enhanced convergence, which makes the deep-convective areas contract—processes consistent with the analysis presented in [18] with regard to the observed ‘deep-tropical squeeze’ in climate model projections. As noted by Jakob *et al.* [19], the tropical atmosphere as a whole is close to a RCE state.

The increase in low-level convergence in the deep-convective ascent areas is more modest in the 100% increase experiment compared to the experiment with a 25% increase in L (figure 7). This can explain why deep-convective areas contract more strongly in the 25% increase experiment than the 100% increase simulation (table 3, highlighted by red colour). Changes in atmospheric stability, which are discussed in the next section, cannot be the reason for the latter behaviour.

### 4.3. The role of atmospheric stability

The atmospheric temperature profiles in the RCE sensitivity experiments vary significantly (figure 5), implying different dry-static stabilities. The larger the specific latent heating L, the more stable the atmosphere. Figure 5 also shows moist adiabats calculated using the corresponding specific latent heat of condensation for the various experiments starting from 800 m height (black lines). Intriguingly, only in the case of the standard simulation, using the actual specific heat of condensation, does the temperature profile agree with the moist adiabat. With increasing L, the atmosphere becomes more stable but remains less stable than the moist adiabat, and vice versa for decreased L. We are unable to say whether this is because the model is tuned to the present-day climate or whether there is a more fundamental reason for this behaviour, but deviations from moist adiabtic lapse rates are also seen in global climate model simulations (e.g. [20–22]). For the computation of the moist adiabatic lapse rate, the formula derived in appendix E of [23] is used. In this calculation of the moist adiabat, a constant dry-adiabatic lapse rate is assumed, which, on a theoretical level, assumes hydrostatic equilibrium. The profiles are calculated based on global mean data and a temporal mean over one month, and the hydrostatic assumption was found to hold well for all sensitivity experiments. The calculation of the moist adiabat is started at 800 m height where all simulations commence to deviate from the dry adiabat. The exact height at which the moist adiabat is initiated has only a relatively minor, and no qualitative, effect on the figure.

The specific latent heat of condensation L is not the same as the actual latent heating occurring in the simulations, a point that we shall return to in the next section. The differing dry-static stabilities have an effect on both the deep-convective and the strong-convective ascent areas. This is reflected in the non-monotonicity of the changes in area fractions. A more stable atmosphere counteracts the stronger latent heating and reduces the occurrence of deep-convective or strong-convective updrafts.

Table 3 reveals some non-monotonic behaviour in the different ascent area fractions (examples are highlighted with red text). One is that the strong-convective area fraction is smaller in the 100% increase experiment than in the 50% increase experiment. Table 5 lists values of total latent heating $Q_{\text{latent}}$ and atmospheric dry-static stability for the different experiments. Total latent heating here is calculated based on mean rainfall and using the corresponding specific latent heat of condensation for the various experiments. Here, $Q_{\text{latent}}$ corresponds very well with the global mean latent heat flux at the surface. To calculate the atmospheric stability, a linear fit was applied to the global-mean, month-mean temperature profile between 1500 m and 10 000 m height.

**Table 5.** Month-mean, global mean latent heating and dry-static stability for the different sensitivity experiments.

experiment	$Q_{\text{latent}}$	stability Γ
standard	120.798	4.2538
25% increase	146.476	5.5377
50% increase	145.859	5.6371
100% increase	146.658	6.0270
25% decrease	109.727	3.3782
50% decrease	85.859	2.1924

Both $Q_{\text{latent}}$ and the dry-static stability are part of the approximate balance equation,

(4.1) $$w\cdot \frac{\partial s}{\partial z}=Q_{\text{latent}}+Q_{\text{radiative,}}$$

where w is vertical velocity and s dry static energy [2]. The equation is based on temperature gradients being weak in the middle troposphere, an assumption that is well met in RCE simulations. Equation (4.1) formulates a balance between vertical velocities or transport, static stability and the diabatic heating terms. More diabatic heating tends to increase the stability and increase vertical velocities. However, increased stability tends to act against upward vertical transport. Therefore, increased diabatic heating will not linearly scale with dry-static stability. Moreover, in the experiments considered here, the specific latent heating of condensation is varied, and it is not immediately clear how this affects $Q_{\text{latent}}$ exactly.

In RCE, global $Q_{\text{latent}}$ balances global $Q_{\text{radiative}}$ and global-mean w is close to zero. Nevertheless, the dry-static stability is related to $Q_{\text{latent}}$ across the different experiments, but not linearly. As with $Q_{\text{latent}}$, the dry-static stability acts as a negative feedback to limit the increase in strong-convective area fraction. So formally one could formulate a feedback equation,

(4.2) $$\alpha_{\text{sc}}\propto F_{\text{specific}\_\text{latent}}-\lambda \cdot \Gamma ,$$

where $\alpha_{\text{sc}}$ denotes the strong-convective area fraction, $F_{\text{specific}\_\text{latent}}$ is the forcing applied by changing the specific latent heat of condensation, Γ is the dry-static stability and λ is a feedback factor. Similarly, the change in $Q_{\text{latent}}$ is muted by the stability Γ. In terms of the ingredients, this is reminiscent of the equation for changes in extreme precipitation under warming by O’Gorman & Schneider [24]:

(4.3) $$c=-\omega \cdot \frac{\text{d}q_{s}}{\text{d}p}{|}_{\text{moist}\_\text{adiabat}},$$

where c is the condensation rate, ω the vertical pressure velocity, $q_{s}$ saturation specific humidity and the derivative of saturation specific humidity with respect to pressure is taken along a moist adiabat. The change in stability is taken into account here by assuming that the atmospheric temperature profile in strong-convective clouds changes according to changes in the moist adiabatic lapse rate. In this context, the role of $F_{\text{specific}\_\text{latent}}$ is taken by low-level moisture which scales approximately according to the Clausius–Clapeyron relation and provides the potential for increased latent heating in strong updrafts under warming.

In the present sensitivity experiments, the dry-static stability turns out to scale rather directly with the changes in the specific latent heat of condensation (table 5). Equation (4.2) does not represent a strict balance, but it replicates the physical processes: the change in specific latent heating is the cause for the differences between the experiments, it leads to changes in $Q_{\text{latent}}$; the resulting alterations in static stability act as a feedback mechanism that affects the changes in strong-convective or deep-convective vertical velocities.

Jeevanjee [25] suggested that the changes of vertical mass flux in the middle troposphere under warming are more appropriately analysed at a fixed temperature instead of a fixed pressure. Because of the differing vertical dry-static stabilities in the sensitivity experiments, a fixed temperature in the free troposphere is achieved at different heights or pressures. The deep-convective and strong-convective area fractions do vary with height and pressure; however, the height dependence of the ascent area fractions is not the same in all experiments, a circumstance again related to the different vertical temperature profiles in the simulations. Unfortunately, for the present simulations, the vertical velocities are not available on temperature levels and therefore we are not able to compute the ascent area fractions at a fixed temperature to the required precision, but it would be an interesting analysis to carry out.

Histograms of vertical velocities at 500 hPa for the different experiments provide more insight into how the distributions of updrafts change (figure 8). The left-hand panel of figure 8 shows how the shapes of the distributions change particularly when comparing the standard experiment with the increased L experiments. In the standard experiment, there are more modest updrafts, but less strong-convective updrafts. By definition of the experiments, the more pronounced strongest updrafts in the increased L experiments are driven by stronger latent heating. The contraction of the deep-convective areas is equivalent to the fact that there are fewer updrafts of modest strengths in the increased L simulations. The increased stability is probably not the main or only reason for the decrease in the deep-convective areas because then the strongest updrafts would reduce, too, which is not the case. So the physical process which mainly leads, or certainly contributes to, the contraction of the deep-convective areas in the increased L experiments is the intensification of low-level convergence into the deep-convective areas. This is also suggested by table 4 which lists mean values of the deep-convective updrafts for the different experiments. The deep-convective updrafts get generally stronger in the increased L experiments even though their areas contract and even though the stability increases in these experiments. Increased stability may, however, contribute to reducing the number of updrafts, and lead to a levelling off of the increase in the strong-convective areas. But the strong-convective uprafts do get stronger in the mean for the increased L experiments compared to the standard simulation (table 4).

**Figure 8.**
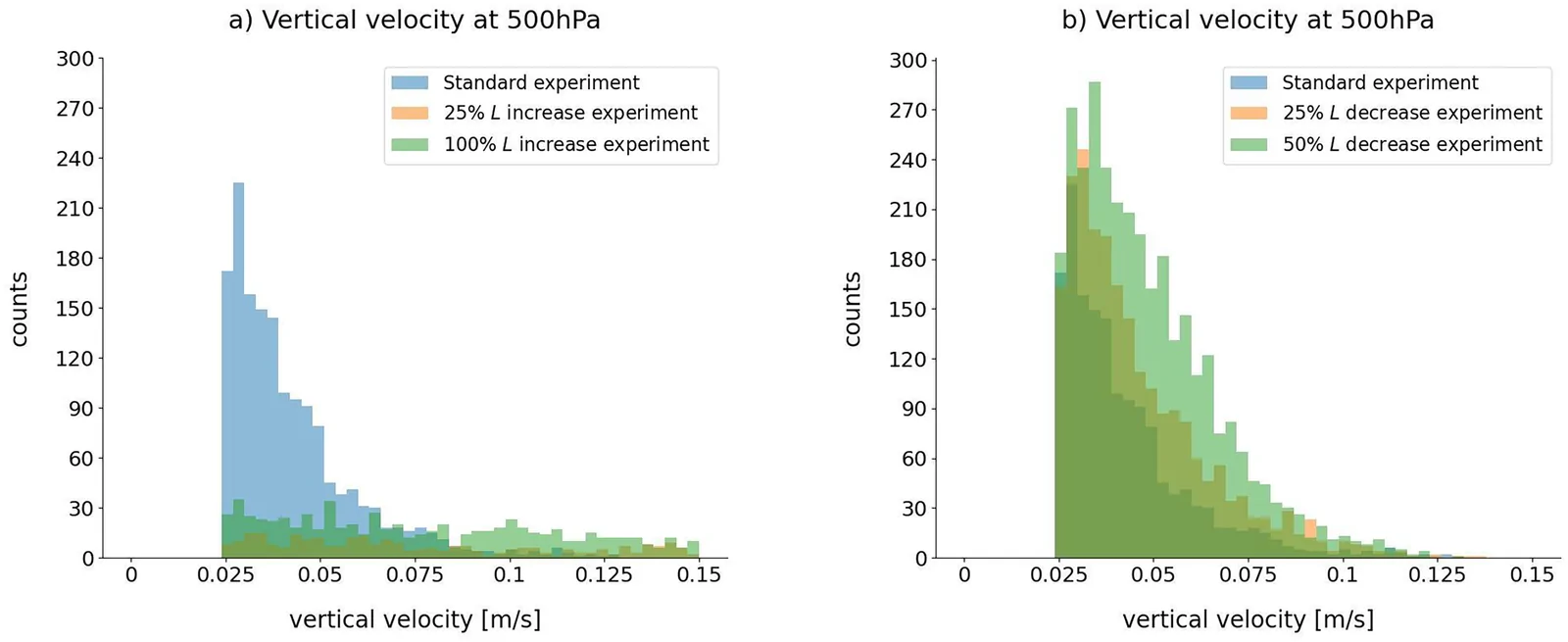
Histograms of vertical velocities, including data from a single time step and only considering vertical velocities greater than 0.025 m s^–1^. The increased latent heating experiments have fewer modest, but more of the strongest updrafts. The decreased latent heating experiments have more modest updrafts but fewer of the strongest updrafts.

The right-hand panel in figure 8 compares the standard simulation with the decreased L experiments. The standard experiment has generally fewer deep-convective updrafts, leading to a smaller deep-convective area, but also a slightly smaller strong-convective area (compare to table 3). In the mean, both the deep-convective and the strong-convective updrafts are of similar strengths in the decreased L simulations compared to the standard experiment (table 4). Both the smaller low-level MSE gradients and the decreased stability probably contribute to larger deep-convective area fractions in the reduced L experiments. That reduced low-level MSE gradients and related convergence are important for the behaviour of the reduced L simulations is indicated by the fact that the deep-convective areas increase quite considerably compared to the standard experiment with roughly equal mean values for the deep-convective updrafts (tables 3 and 4). The effect of the stability is that despite significantly reduced latent heating, the mean strength of the deep-convective and strong-convective updrafts are similar in the reduced L experiments compared to the standard simulation.

### 4.4. The atmospheric energy budget

To better understand the behaviour of the different sensitivity experiments and how it relates to radiative fluxes, it is instructive to consider the global atmospheric energy budget across the different simulations (figure 9). The cause of the differences between the various RCE sensitivity experiments presented here is the changed specific latent heat of condensation, and the various terms in the energy budget adjust to this change. So for the presented sensitivity experiments, it would be misleading to *explain* the observed changes in the circulation with changes in the fluxes that enter the atmospheric energy budget.

**Figure 9.**
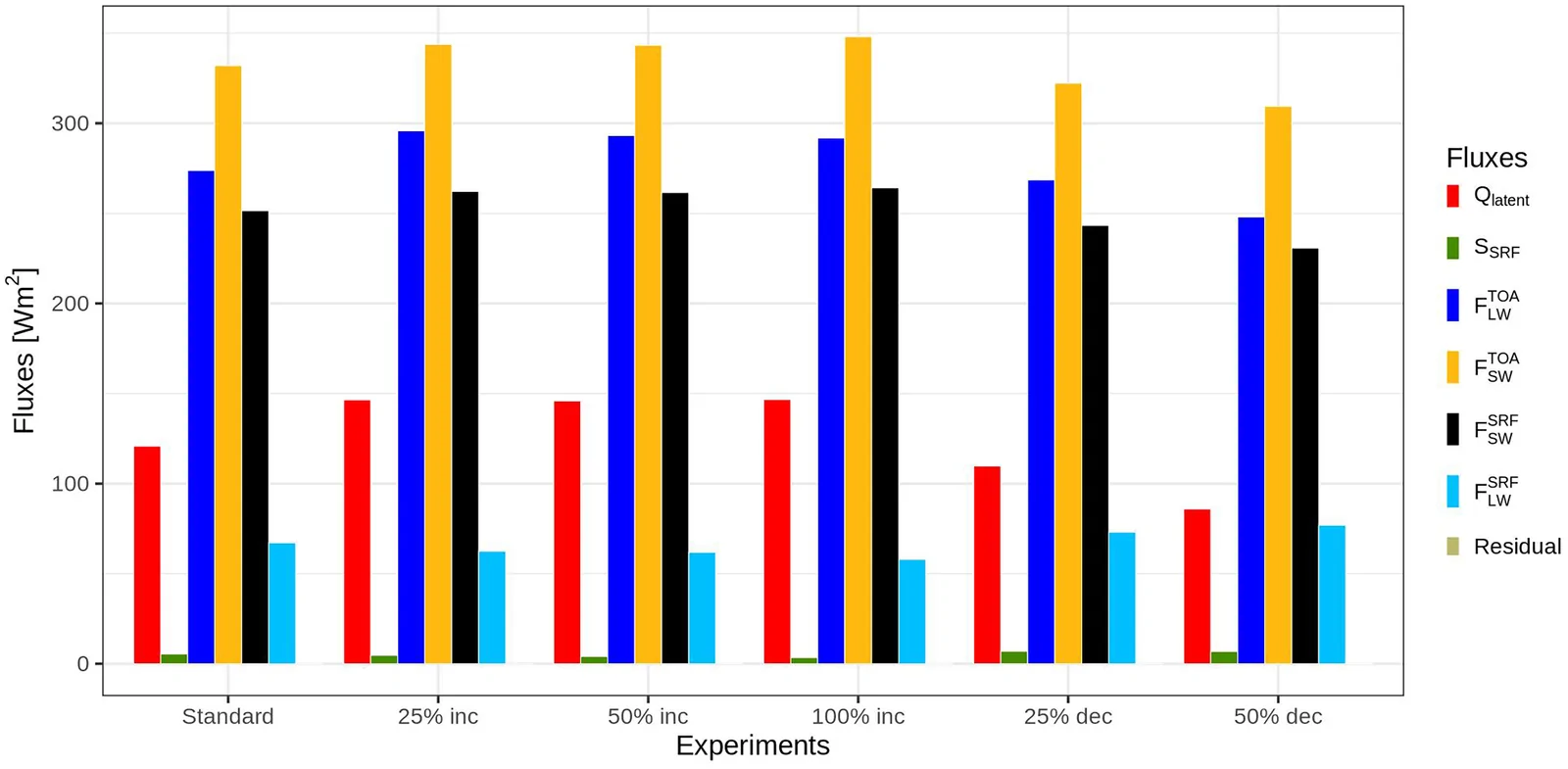
The various terms in the atmospheric energy budget for the different experiments. For the figure, all terms are presented with a positive sign. The last column includes the residual in the energy budget equation (4.4); for the calculation of the budget, a positive sign is assumed to represent fluxes directed into the atmosphere.

The atmospheric energy budget equation is (for more details see [26]):

(4.4) $$Q_{\text{latent}}=S_{\text{SRF}}+Q_{\text{radiative}}=S_{\text{SRF}}+F_{\text{SW}}^{\text{TOA}}+F_{\text{SW}}^{\text{SFC}}+F_{\text{LW}}^{\text{TOA}}+F_{\text{LW}}^{\text{SFC}},$$

where the radiative fluxes are divided into TOA and surface (SRF) fluxes and LW and shortwave (SW) fluxes. Here, $S_{\text{SRF}}$ denotes the sensible heat flux at the surface. Fluxes into the atmosphere are defined to be positive in the calculation of the budget.

The first bar in figure 9 repeats the latent heating terms (also) included in table 5. It differs from the changes in the specific latent heating mainly in that the effective global latent heating is very similar in all the increased L experiments as a result of a balance that is established. The sensible heating at the surface (second bar in figure 9) is generally smaller and varies less than other terms. It tends to decrease in the increased L experiments and increase in the decreased L simulations because the atmosphere warms or cools, respectively. This is also part of the reason why the OLR at the top of the atmosphere changes, but OLR also depends on the vertical temperature structure, humidity and clouds. OLR is the result of a complex balance and it is obvious that a single term like OLR cannot be called the cause of the observed changes or be used to *explain* the behaviour of the different sensitivity experiments. OLR tends to behave rather similarly to $Q_{\text{latent}}$, but this is by no means obvious *a priori*. To some extent the TOA net SW radiative fluxes also replicates the changes in OLR, albeit not perfectly. In the increased L experiments, the net SW fluxes at the top of the atmosphere increase quite considerably, and vice versa in the decreased L experiments (increased SW flux here means less reflected SW flux). So to some extent, the changed OLR is balanced by the changes in TOA SW fluxes, an effect that is also observed in the global kilometre-scale warming experiments described in [12]. This implies that, to a certain extent, the changed atmospheric latent heating is balanced by changed radiative fluxes at the surface. The changed radiative fluxes at the surface would alter the surface temperature if the surface temperature was not fixed. But ultimately the exact changes in the various terms are the result of complex balances and cannot be explained based on fundamental, first principles. A process-based view on the changes, however, provides a better understanding of the physical mechanisms involved.

### 4.5. Available potential energy production

In [27], a useful perspective on the analysis of the role of different diabatic processes in the context of climate simulations was introduced—the consideration of the APE budget. The interesting aspect about focusing on which physical mechanisms generate most of the APE is that it highlights the processes which generate the atmospheric circulations, a key aspect in questions related to weather and climate. In the present context, it helps to understand which processes contribute to changing the convective ascent areas through dynamical processes across the different sensitivity experiments. It is a distinctly different view from the perspective gained from an MSE budget analysis, for example. MSE is approximately invariant following air parcels as they undergo phase changes between water vapour and liquid, therefore somewhat obscuring the role of latent heating in the coupling between moist convection and the circulation and highlighting radiative and surface processes. Ultimately, circulations are created because of the existence of APE, so the question which processes contribute most to the creation of APE is of interest.

We shall not discuss the full equation for the APE budget given in [27] here. The consumption of APE mainly happens through conversion to kinetic energy on a wide range of scales. The most important term in the present context is the term which describes the production of APE by various physical processes:

(4.5) $$\text{production of APE}=\int_{0}^{z}\frac{\rho^{\text{tm}}}{N^{2}}\;\overset{―}{b\cdot S_{b}}\text{d}z^{\prime },$$

where $\rho^{\text{tm}}$ is a reference density, here taken to be the global-mean, time-mean density, b is the buoyancy, $S_{b}$ the production of buoyancy by various different physical processes and $N^{2}$ the square of the Brunt–Vaisala frequency. The buoyancy b is defined as

(4.6) $$b=g\cdot \left(\frac{\theta^{\prime }}{\bar{\theta }}+\frac{\epsilon \cdot q^{\prime }}{1+\epsilon \cdot \bar{q}}\right),$$

where g=9.81 m s^–^${}^{2}$ is the gravity acceleration, q is specific humidity, θ potential temperature, the overbar denotes the global mean and a prime the deviation from the global mean. The computations use six-hourly fields; the constant ϵ=0.61. The buoyancy and the corresponding buoyancy tendencies of the different physical processes thus have a temperature component and a humidity component. The calculation is done for one month of data at the end of the 3-year simulations and on model levels. For each physical process, the buoyancy tendencies are

(4.7) $$S_{b}=g\cdot \frac{S_{\theta }}{\bar{\theta }}+g\cdot \frac{\epsilon S_{q}}{1+\epsilon \bar{q}},$$

where $S_{\theta }$ is the potential temperature tendency and $S_{q}$ is the respective specific humidity tendency. The APE production terms are calculated for various physical processes at every six-hourly time stamp, and the mean over one month is computed at the end. The largest terms in the APE production are the ones which include only temperature tendencies in equation (4.7). The ones which are based on the moisture tendencies are typically an order-of-magnitude smaller than the corresponding production terms derived from the temperature tendencies. For the standard experiment and the increased L experiments, figure 10 shows the APE production from temperature tendencies of various different physical parametrizations in the model: convection, LW radiation, SW radiation, microphysics and the turbulence scheme.

**Figure 10.**
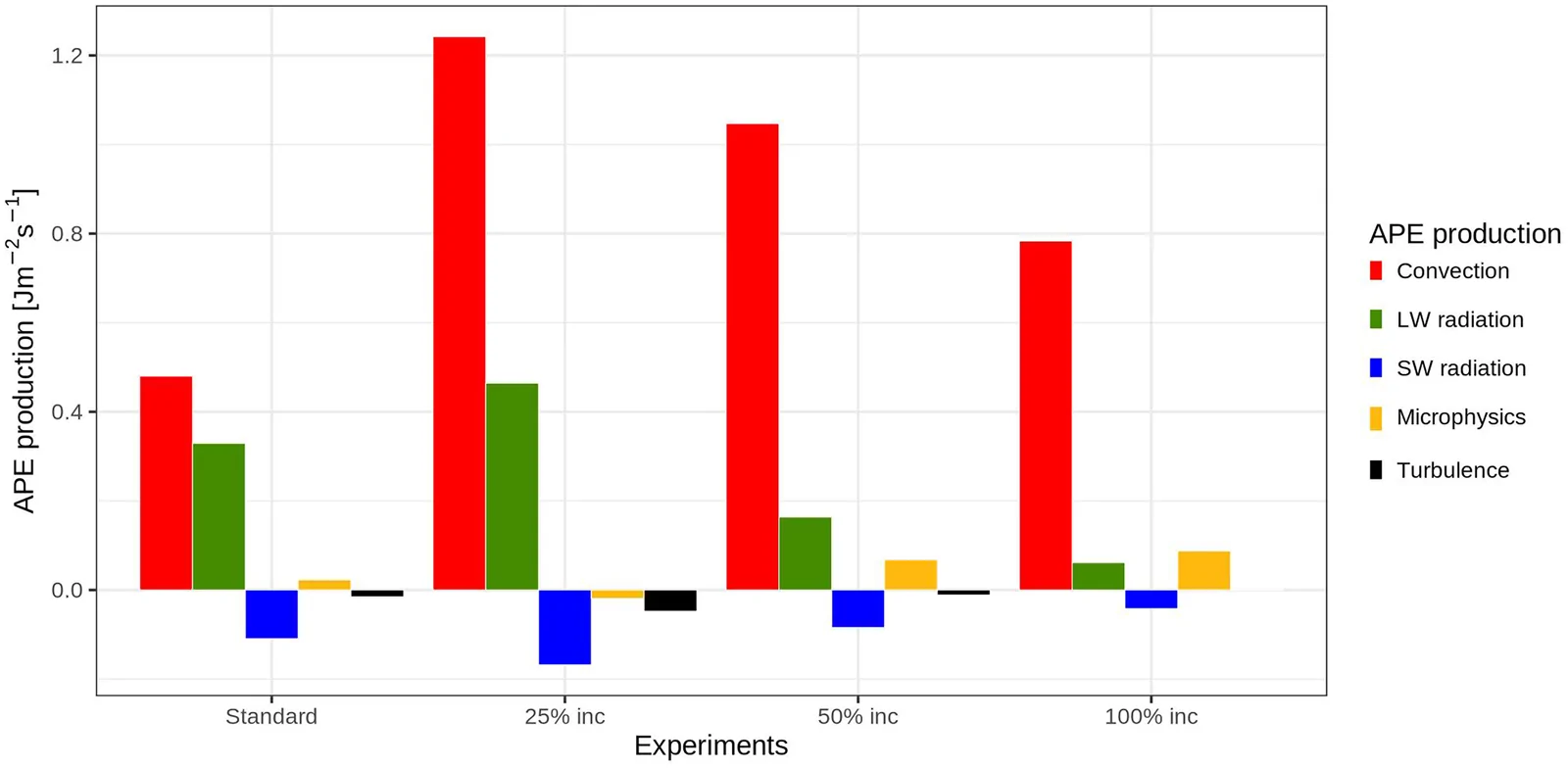
APE production owing to the temperature tendencies of different physics schemes for some of the RCE sensitivity experiments.

The temperature tendency from convective latent heating is the most important term which generates APE in all experiments; it is generally more important than radiative processes. This is significant because it provides a different view on the role of phase transitions and related latent heating in the context of understanding the circulation response of the different sensitivity experiments. The APE budget reflects the fact that it is the convective latent heating which primarily leads to the changes in the circulation characteristics across the different experiments and directly generates the necessary APE. While convective motion tends to consume APE and adjust the atmospheric profile to a neutral state, the positive correlation between buoyancy and buoyancy tendency from latent heating (see equation (4.5)) can be seen as an important source generating APE and thus causing this atmospheric motion. Note that the LW tendency becomes small in the 100% increased L experiment which is consistent with the strong contraction of the deep-convective area in the doubled L experiment. By contrast, the role of convective heating becomes even more pronounced in the increased L simulations compared to the other processes.

The particularly pronounced role of the convective temperature tendency in the 25% increased L experiment in generating APE reflects the strongest contraction of the deep-convective area fraction in this simulation. This again suggests that dynamical processes, namely the enhanced convergence into the deep-convective areas, are a key mechanism in making the deep-convective areas contract and related to the creation of APE by moist convection in these regions. The changes in the strong-convective ascent areas are a more direct, local response to the varied specific latent heating, and the feedback from the atmospheric stability has more of an influence in this case.

The different terms in the budget have spatial characteristics as expected. Figure 11 exemplifies the spatial structures for the APE production terms related to temperature tendencies of convection and LW radiation. Snapshots for one particular time at a vertical level of 1500 m height in the reference experiment are shown for different related quantities. For context, the OLR field is also shown in figure 11a and the buoyancy b in figure 11b. Figure 11c,d shows buoyancy production from the temperature tendency of convection and LW radiation, respectively. Figure 11e,f includes the global average of buoyancy times buoyancy production of the two processes, part of the integrand in equation (4.5). APE production of convective latent heating is strong where deep convection occurs, and APE production of LW radiation is concentrated in clear-sky or low-cloud areas where radiative cooling is significant.

**Figure 11.**
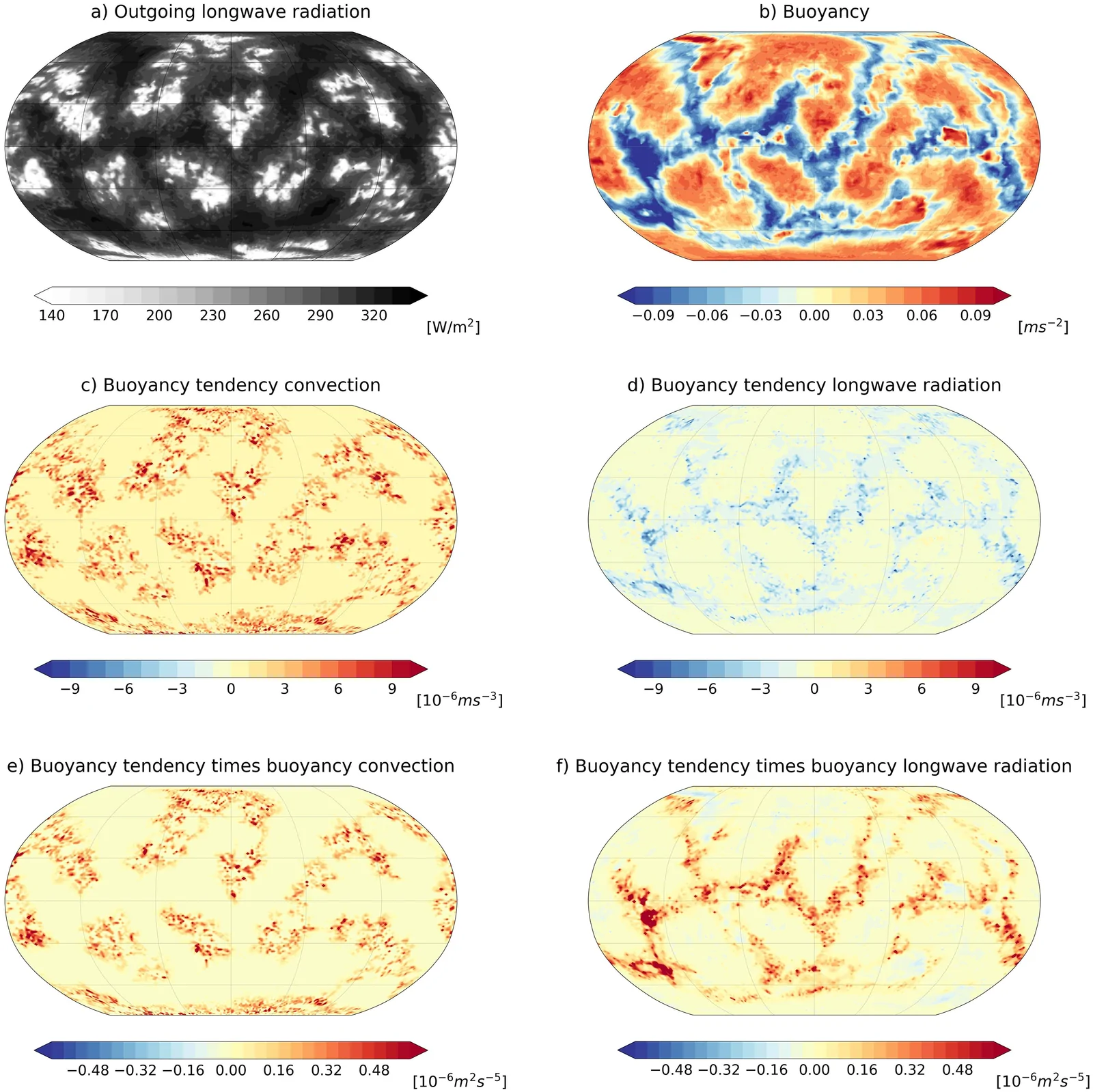
All plots are for the standard experiment. Panel (a): OLR panel (b): buoyancy at 1500 m, panel (c): buoyancy tendency from temperature tendency of convection at 1500 m, panel (d): buoyancy tendency from temperature tendency of LW radiation at 1500 m, panel (e): buoyancy tendency from temperature times buoyancy from convection at 1500 m and panel (f): buoyancy tendency from temperature times buoyancy from LW radiation at 1500 m.

## 5. Discussion and conclusions

Explanations of changes in the tropical circulation under warming are often based on an energy budget framework and involve referring to changes in radiative fluxes. In [2], the contraction of the tropical ascent area under warming is explained by the fact that the latent heating in the ascent region intensifies faster than the increase in the radiative cooling in regions of descent. The argument thus in part relies on how radiative fluxes change under warming. A similar reasoning is commonly used when explaining future changes in rainfall. The increase in rainfall under warming is more moderate than that suggested by the Clausius–Clapeyron relation because the TOA OLR increases more slowly than the Clausius–Clapeyron scaling [8]. However, we do not have a fundamental physical theory of how OLR or other TOA radiative fluxes will change under global warming. OLR is part of the TOA energy budget, among various other terms. How OLR changes is a consequence of changes in fundamental moist processes and quantities such as the distribution of specific humidity, clouds and the vertical structure of the atmosphere. Explaining the circulation changes with how radiative fluxes at the top of the atmosphere change can lead to a shift of the focus away from some of the key and fundamental moist processes. Such a perspective can suggest that moist processes are a consequence of radiative flux changes, a view that is debatable as the TOA radiative flux changes are not necessarily external forcings but may be a consequence of the changes in circulations, moisture and clouds within the Earth system, at least in the context of natural weather and climate variability.

Starting from the observation that in different datasets, including ERA5 reanalysis and global kilometre-scale warming simulations, the deep-convective area fraction decreases in the tropics under warming and the strong-convective area fraction increases, in the present article we highlight the importance of moist dynamical processes in these changes of the tropical circulation. To this end, we use RCE simulations with a varying specific latent heat of condensation as a controlled experimental set-up to exemplify this perspective. In these sensitivity experiments, the physical cause of the emerging changes is known by design, namely the *a priori* change in the amount of specific latent heating. The change in specific latent heating does not linearly translate into the variation of the total global latent heating in the simulations, and the modification of the specific latent heat of condensation does not map to changes in the circulation characteristics in a straightforward way because of two key feedbacks. One of the feedbacks is the change in the dry-static stability of the atmosphere, and the other is a variation in the low-level convergence and moisture convergence. These two feedbacks are immediate consequences of the change in specific latent heating and can be understood from a process-based perspective.

The expansion of the strong-convective areas is a direct consequence of the varied latent heating as the latent heating directly drives the circulations and in particular the most extreme updrafts. However, the resulting altered dry-static stability will at some point affect even the most extreme updrafts. The reduction of the deep-convective area fraction is mainly a consequence of the stronger low-level convergence under increased specific latent heating and low-level MSE gradients which leads to a contraction of the deep-convective areas. The atmospheric stability also comes into play. For example, in the L decrease experiments, the deep-convective areas expand quite considerably, which can in part be due to the generally decreased stability.

The RCE sensitivity experiments considered here are not global warming experiments, but there is an analogy. Under surface warming, the low-level specific humidity tends to increase [28,29] following the increase in the near-surface temperature [30] (figure 12). As a consequence, when moderate or strong convection happens, the latent heat release increases compared to a no-warming scenario [24]. The increase in near-surface specific humidity does not directly imply more frequent or less frequent convection, but it tends to increase the amount of latent heat release when and where tropical deep convection occurs, and there is evidence that strong convective updrafts intensify under warming [31].

**Figure 12.**
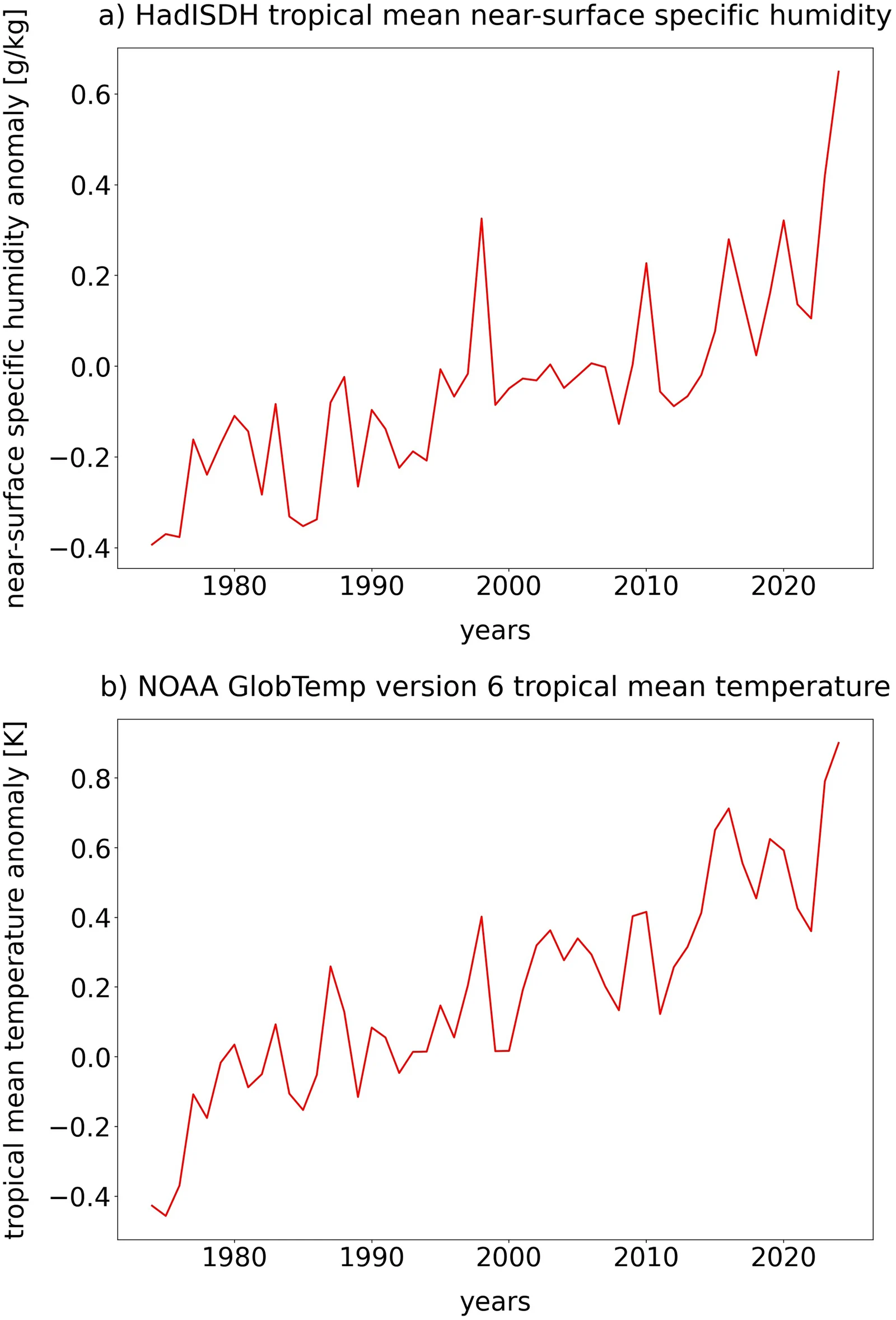
Upper panel: tropical near-surface specific humidity anomaly [28,29]. The reference period is 1991 to 2020. Lower panel: tropical near-surface temperature anomaly [30]. The reference period is 1971 to 2000.

In a more process-based perspective and on shorter time scales, radiative fluxes are often not of leading-order importance and can be better understood as being a result or adjusting to fundamental moist dynamical processes. Even though cloud-radiative effects, for example, can amplify or dampen certain features such as the Madden–Julian oscillation, they are obviously the consequence of the processes that lead to cloud formation and many simplified, conceptual models of phenomena of the tropical atmosphere do not include cloud-radiative effects or even interactive radiation [14,17,32–34]. Recent satellite observations also suggest that in deep-convective clouds, latent heating is roughly an order-of-magnitude larger than radiative effects [35].

The important role of moist processes and latent heating is also highlighted by the fact that it is latent heating in convection that generates most of the APE in the RCE sensitivity experiments. It is this APE which ultimately leads to and drives the circulations that contribute to changes in the characteristics of the deep-convective and strong-convective area fractions.

In their conclusions, Jenney *et al.* [2] highlight the importance of better understanding moist processes in the tropical ascent areas and the perspective advocated in the present article does not contradict their study. Even though Jenney *et al.* [2] adopt an energy budget framework as a starting point, they also come to the conclusion that further studying the moist-dynamical processes in the deep-convective areas is necessary for making continued progress in understanding the changes in characteristics of the ascent areas under warming.

What the RCE sensitivity experiments show is that the changes in deep-convective and strong-convective ascent areas, to first order, scale with the resulting global latent heating $Q_{\text{latent}}$. Here, $Q_{\text{latent}}$ is, however, not linearly related to the prescribed specific latent heating. It is, as the ascent area fractions, affected by two main feedbacks, namely the effects from atmospheric stability and low-level convergence. The atmospheric stability is itself a result of changed latent heating and can therefore most appropriately be understood as a feedback mechanism. It is not defined purely by moist adiabatic lapse rates; the profiles in the sensitivity experiments diverge from moist adiabatic profiles when the specific latent heating is changed away from the actual value. The effect of changed low-level MSE gradients is at least partly a dynamical effect in that it is a result of increased low-level convergence into the areas of deep convection. The areas around the constricted deep-convective regions are still relatively moist and the dry areas do not expand pronouncedly (figure 4). This is consistent with the analysis of convective organization in RCE by Bretherton *et al.* [36] showing that the areas around organized deep convection are relatively moist, at least at lower levels, and covered by shallow clouds, a circumstance that supports deep-convective self-aggregation. The direct effect of increased specific latent heating is amplified by convection-circulation-moisture feedbacks as for example described in moisture-wave instability by Jiang *et al.* [37] on synoptic scales or the intensification of tropical storms by latent heating on mesoscales [38].

In the present article, we advocate a more process-based view on changes in tropical circulation characteristics under warming. It helps to focus on key moist processes and is distinctive from an energy budget approach which identifies energetic balances. In particular, it suggests that radiative effects can in many situations more appropriately be interpreted as adjusting to moist dynamical processes and latent heating instead of the latent heating being a consequence of radiative effects [39]. In that perspective, convective-radiative equilibrium could be seen as an interaction between convective latent heating, resulting circulations and alterations in atmospheric stability. In the often more transient and unbalanced situations of real-world tropical weather and climate, this is even more so the case. Of course, latent heating and moisture are not akin to an external forcing as in the RCE sensitivity experiments. In the climate system, all processes interact and there are different possible perspectives on this complex system. The moisture dynamics perspective is, we would argue, however, close to the important processes that shape the manifold different phenomena in the tropical atmosphere. The view also highlights the need for further research efforts that specifically target a better understanding of moist dynamical processes, such as cloud microphysics or convective vertical motions, which are generally difficult to observe. Future Earth observation missions [40–42], field campaigns and new high-resolution reanalyses will deepen our understanding of the important moist processes and their coupling to the atmospheric circulation which shape tropical weather and climate on a wide range of scales.

## Data Availability

ERA5 data [11] were downloaded from the Copernicus Climate Change Service (C3S) Climate Data Store (http://cds.climate.copernicus.eu/cdsapp#!/dataset/reanalysis-era5-pressure-levels). For the availability of the HadISDH near-surface humidity dataset, see [28] and [29]. The NOAA GlobTemp version 6 dataset is referenced and documented in [30]. The scripts to reproduce the figures in the present article are publicly available at [43].
